# The availability and functional status of focused antenatal care laboratory services at public health facilities in Addis Ababa, Ethiopia

**DOI:** 10.1186/s13104-016-2207-z

**Published:** 2016-08-11

**Authors:** Daniel Melese Desalegn, Serebe Abay, Bineyam Taye

**Affiliations:** 1 Addis Ababa Public Health Research and Emergency Management Core Process, Addis Ababa City Administration Health Bureau, Addis Ababa, Ethiopia; 2 Technology Transfer and Research Translation, Ethiopian Public Health Institute (EPHI), Addis Ababa, Ethiopia; 3 School of Public Health, College of Health Sciences, Addis Ababa University, Addis Ababa, Ethiopia

**Keywords:** Focused antenatal care laboratory service, Availability, Functional status, Addis Ababa, Ethiopia

## Abstract

**Background:**

Provision of quality laboratory services is an essential aspect of a promoting safe motherhood and better outcomes for newborn. Therefore; this study was intended to assess status of focused antenatal care (FANC) laboratory services at public health facilities in Addis Ababa, Ethiopia.

**Methods:**

Institution based, descriptive cross-sectional study was conducted from April to May 2015. The study included 13 randomly selected health facilities and 13 purposively selected laboratory service providers. The status of FANC laboratory service was assessed by using pre-tested structured questionnaire and observation checklist. The study supplemented with qualitative data through in-depth interview of laboratory service providers. The quantitative data were coded and analysed by using SPSS Version 20 software and qualitative data was transcribed, coded, categorized and thematically analysed by the principal investigator.

**Results:**

Only 5 (38.5 %) out of 13 visited health facilities reported the availability of all types of basic FANC laboratory investigations. Comparing the availability of individual tests in the study facilities, urine dipstick, urine microscopy and stool examination were available in all institutions. However, only 7 (53.8 %) of the health facilities reported the availability of hepatitis B virus screening test. Rapid syphilis (RPR) test was found in 10 (76.9 %) facilities. All laboratory facilities had at least one or more basic FANC laboratory tests interruption for more than a day within the last 1 year due to shortage of reagent and electric power disruption.

**Conclusions:**

Majority of the health facilities reported incomplete provision of FANC laboratory investigations. Laboratory supply shortage and electric power disruption were the facilities’ major challenge to screen pregnant women for pregnancy related health conditions. Since such conditions may affect the outcome of pregnancy, therefore extensive efforts should be targeted to avoid services interruption by taking improvement measures including the fulfilment of all FANC laboratory resources.

## Background

Most maternal deaths are preventable, as the health care solutions to prevent or manage the complications are well known. This includes well-functioning health system that provides high quality care in the health facility level [1]. In order to decrease maternal morbidity and mortality, strong health systems, accessible, available and satisfactory cares are needed. Particularly laboratory services continue to play a critical role in all disease control and prevention programmes by providing timely and accurate information for use in patient management and disease surveillance [2].

World Health Organization (WHO) recommends a minimum of four goal oriented FANC visits during a woman’s pregnancy. At minimum, haemoglobin (Hgb) and testing of urine for albumin and sugar are recommended in every FANC visit and tests for syphilis, HIV status as well as blood group and rhesus (Rh) factor are recommended at least once. FANC visits also includes examination for signs of chronic conditions and infectious diseases such as HIV, malaria, syphilis and other sexual transmitted infection (STIs), anaemia, heart disease, diabetes, malnutrition, and tuberculosis (TB). Such conditions may affect the outcome of pregnancy and therefore, requires immediate treatment, and intensive monitoring and follow-up throughout the course of pregnancy [3, 4].

Provision of quality laboratory services is an essential aspect of a promoting safe motherhood and better outcomes for newborns, however, as a result of paucity of funds, irregular power supply, limited technology, administrative bottlenecks and lack of human resource capacity in clinical laboratories are a major barrier to test utilization, due to these several healthcare facilities are without functioning medical laboratories in Africa [5]. Laboratory service in Ethiopia is very weak and the quality assurance system practiced in the supervised laboratories was generally either very weak or non-existent, except for HIV screening tests, no quality assessment scheme was established for other tests [6].

Millennium development goal (MDGs) 2012 report finds, providing incomplete or poor quality maternal service in Ethiopia is a major challenge to achieve maternal mortality reduction goal regardless of the increased extensive efforts to improve maternal health [7]. The recent Ethiopia Mini Demographic and Health Survey (EDHS) 2014 report showed that, among women with antenatal care, nearly 41 % of the ANC clients did not have their urine test and 35 % did not have their blood test [8]. This implied identification of pre-existing health conditions that may affect outcome of pregnancies such as HIV, anemia and other sexually transmitted infections were not provided. Such missed laboratory tests are indicators of poor quality FANC services [9].

The Ethiopia Federal Ministry of Health report showed that, among pregnant women who have received antenatal care, only 75.5 % of them were tested for HIV [10]. Mothers without ANC follow up were five times more likely to have an infant with HIV sero positivity than those who had ANC visits, but not all the recommended FANC procedures were carried out for every woman, at every healthcare facility due to lack of some specific laboratory services [11, 12]. Similar study done to assess the status of HIV laboratories in Ethiopia showed that, majority of laboratories (62 %) do not follow a specific testing algorithm and 29 % do not confirm their results at all [13]. Other study showed that due to lack of properly follow test procedure, shortage of supplies and laboratory equipment, absence of effective laboratory equipment maintenance, lack of water, unreliable electricity and substandard designed laboratory rooms are major clinical laboratory problems in Ethiopia [6, 13].

Challenges on maternal health are complex, it require researches to identify the bottle necks in each level of maternity health services. This study will provide over view on the current status of FANC laboratory services in Addis Ababa public health facilities and our findings will identify factors which will be useful to improve the FANC laboratory services delivered by the health system of the study area in particular and other similar setting. It also could create an opportunity for the administrators of public health facilities to take corrective action early.

## Methods

### Study setting and context

Institutional based, descriptive cross-sectional study was conducted between April and May 2015. The study was conducted in Addis Ababa which is the capital city of Ethiopia. Addis Ababa has 10 sub-cities and 99 districts and this city is found at an average altitude of 2400 meters (about 8000 ft.) above sea level with an estimated area of 530.14 km^2^ [14, 15]. According to the 2007 national census, Addis Ababa has a population size of 2.74 million with annual growth rate of 2.1, of which 52 % were females and 34.4 % were women in a reproductive age [14]. In Addis Ababa, there were a total of 45 hospitals and 74 health centres. Of the 45 hospitals, 28 were privates, 10 were publics, 4 are non-governmental organizations and 3 are armed forces (military) hospitals [14, 15]. In Ethiopia a regional hospital expected to serve 1–1.5 million populations, while a health centre for 25,000 populations. Both health facilities expected to provide FANC services, but health centres are the front line for health services while hospitals in most of the time received clients which refer from health centres for better care and management.

Regarding FANC services, at the time of this assessment, only six hospitals and 74 health centres which providing FANC service at public health facilities in Addis Ababa. This study conducted on 10 health centres and three public hospitals which have been providing FANC services for pregnant mothers. The service render in these health facilities for FANC laboratory services including blood group, Rh, Hgb, HIV, urine test for sugar, keteon and albumin, stool examination for intestinal parasite, rapid syphilis (RPR), hepatitis B virus screening, blood glucose and other laboratory tests. Such tests used for the screening of pregnancy related health problems and intensive monitoring and follow-up throughout the course of pregnancy.

### Study subjects

Thirteen selected public health facilities laboratory, provide focused antenatal care services and 13 laboratory heads (one from each selected health facilities).

### Sampling procedures

Three hospitals and ten health centers (one health center from each sub city) were selected by simple random sampling technique. For the qualitative component of the study, 13 laboratory services providers (one from each selected health facilities) were purposively selected for interviewing based on the purpose and the need of issue being raised.

### Measurement and data collection

Data was collected by using pre-tested structured questionnaire tool. The questionnaire section that included: general information about the facilities, including access to water and power supply; information on type of FANC laboratory services provided, including availability of human resources, essential laboratory equipment and supplies, quality assurance (QA) protocols for FANC laboratory diagnostics and status of biosafety and practices. In addition we used a check list for other observed variables and in-depth interview of laboratory service providers. The collected data were checked for completeness and consistency, and double entered into a SPSS 20.0 (SPSS Inc. Chicago, USA) database. The quantitative data were coded and analysed by using SPSS Version 20 software and qualitative data was transcribed, coded, categorized and thematically analysed by the principal investigator.

### Data quality assurances

The questionnaire was pre-tested over 5 % of the sample size in two health facilities (one hospital and health centre) out of the study site to ensure that it was clear for respondents. After pre-test, some modification of the questioner was made for unclear and difficult question. These pre-test data were not included in the analysis of this study.

Training was given for data collectors and supervisors by the principal investigator to clarify how to collect data. The completeness of the questionnaire was rechecked by supervisors at the end of the day. This was also double checked by the principal investigator.

### Operational definition

#### Basic FANC laboratory services

If health facilities equipped with FANC laboratory tests [blood group, rhesus status (Rh), Hgb, HIV, urine test for sugar, keteon and albumin, stool examination for intestinal parasite, rapid syphilis (RPR) test, hepatitis B virus screening test and blood glucose test] provide for pregnant mothers during focused antenatal care visit.

#### Laboratory services availability

Availability of basic FANC laboratory services in selected health facility during study period.

#### Services functionality

Status of basic FANC laboratory services uninterrupted for more than a day with in the last 1 year.

### Ethical consideration

Before the research work, ethical clearance was obtained from the Departmental Research and Ethics Review committee (DRERC) of school of allied health science department of medical laboratory science, Addis Abba University and from Addis Ababa city administration health bureau. In addition, permission was obtained from respective health facilities. At the time of data collection, a verbal consent was obtained from study participants prior to the study. Moreover, all the study participants were informed verbally about the purpose and benefit of the study along with their right to refuse. Furthermore the study participants were reassured for confidentiality.

## Results

### Facilities and infrastructure

From the total 13 visited laboratory facilities that were involved in this study, 3 (23.1 %) were hospitals and the remaining 10 (76.9 %) were health centers. The study response rate was 100 %.

In this study, health facilities clients’ waiting area and laboratory blood drawing rooms were assessed. All the health facilities had clients’ waiting areas and 9 (69.2 %) of them had clean and separate phlebotomy rooms. In all visited health facilities latrine services were unhygienic, mainly in the hospitals. It had bad odor and the numbers of the toilet were not proportional with clients. None of the health facility laboratories had a place in blood drawing room to put clients’ personal materials (jacket, bag, etc.) during blood specimen collection.

Although all visited laboratory facilities had access to electricity, 7 (53.8 %) of them had faced electric power disruption at least once per day. A participant in the in-depth interview said, “*Sometimes due to electric power disruption we discarded the collected specimen from clients and when power maintain ask mothers to repeat specimen for some specific test like urine and stool because we don’t have standby generators or specimens preservative mechanisms, therefore, unreliable electric power supply were affecting over all our services and clients’ loss trusts on the quality of laboratory services”.* (Female, age 29, laboratory technologies).

The availability and functionality of back-up electric power supply (standby generator) among visited health facilities were variable, 8 (61.5 %) laboratory facilities had standby generators, in 4 (50 %) health facilities the generators did not functional, while in 5 (38.5 %) health facilities generator were not totally available.

Among all visited health facility laboratories 11 (84.6 %) had adequate water supply; but 6 (46.2 %) of them had faced water access interrupted at least once per day. Facilities reporting interruption of water and electric power services were affecting the FANC laboratory services (Table 1).

**Table 1:** Laboratory facility and safety assessment at public health facilities, in Addis Ababa, Ethiopia

Variable	Frequency (n)	Percentage (%)
Having clients’ waiting areas	13	100
Having separated phlebotomy room	9	69.2
Having a place in phlebotomy room to put clients’ material	0	0
Facilities have access to electricity	13	100
Having back-up electric power supply (generator)	8	61.5
Having function back-up electric power supply (generator)	4	30.8
Facilities faced electric power disruption in the last 1 year	7	53.8
Having adequate water supply	11	84.6
Water access interrupted at least once per day in the last 1 year	6	46.2
Having hand washing area	9	69.2
Having separate sinks for washing laboratory wares and staining	8	61.5
Latrine services availability	13	100
Numbers of the toilet proportional with clients	0	0
Latrine cleanness	0	0

April to May, 2015 (n = 13)

### Biosafety

During the survey, laboratory staffs were observed wearing protective gloves and coats in all laboratory facilities and no shortage of personal protective equipment was documented in all assessed facilities. Among the surveyed facilities, hand washing area was observed in 9 (69.2 %), and 8 (61.5 %) had separate sinks for washing laboratory wares and staining (Table 1).

All visited laboratories were used sharp boxes to dispose contaminated sharps and incinerators for solid waste. Liquid waste coming out of the laboratory were released to the sewerage system without any pre-treatment with chemicals or other sterilization techniques.

### Availability of basic FANC laboratory tests

Out of 13 visited health facilities only 5 (38.5 %) were found to provide all types of basic FANC laboratory investigation and the rest 8 (61.5 %) did not provide one or more basic FANC laboratory tests during the study period.

Comparing the availability of individual tests in the study facilities, urine dipstick, urine miscopy and stool examination were available in all institutions. However, only 7 (53.8 %) of the facilities reported the availability of hepatitis B screening test. HIV, RPR and blood glucose tests were available in 11 (84.6 %), 10 (76.9 %) and 11 (84.6 %) respectively, whereas blood group, Rh and haemoglobin tests were available in 12 (92.3 %) visited health facilities (Table 2).

**Table 2:** Availability and functional status of focused antenatal care laboratory services at public health facilities in Addis Ababa, Ethiopia

FANC laboratory Testes	Type of health facility	Availability of FANC test	Functionality of FANC test
		Frequency (%)	Frequency (%)
Blood group and Rh test	Hospital	3 (100)	3 (100)
	Health center	9 (90)	8 (80)
	Total	12 (92.3)	11 (84.6)
Haemoglobin test	Hospital	3 (100)	2 (66.7)
	Health center	9 (90)	6 (60)
	Total	12 (92.3)	8 (61.5)
Urine dip stick	Hospital	3 (100)	3 (100)
	Health center	10 (1000	8 (80)
	Total	13 (100)	11 (84.6)
Urine microscopy	Hospital	3 (100)	3 (100)
	Health center	10 (100)	5 (50)
	Total	13 (100)	8 (61.5)
Stool examination	Hospital	3 (100)	3 (100)
	Health center	10 (100)	5 (50)
	Total	13 (100)	8 (61.5)
Rapid syphilis test	Hospital	3 (70)	1 (33.3)
	Health center	7 (70)	4 (40)
	Total	10 (76.9)	5 (38.5)
HIV test	Hospital	3 (10)	2 (66.7)
	Health center	8 (80)	6 (60)
	Total	11 (84.6)	8 (61.5)
Hepatitis B screening test	Hospital	1 (33.3)	1 (33.3)
	Health center	6 (60)	5 (50)
	Total	7 (53.8)	6 (46.2)
Blood glucose test	Hospital	3 (100)	3 (100)
	Health center	8 (80)	6 (60)
	Total	11 (84.6)	9 (69.2)

n = 13(health centre = 10, hospital = 3)

April to May, 2015

Comparing the availability of tests in the hospitals and health centres, blood group and Rh, RPR, blood glucose, haemoglobin and HIV tests were available in all hospitals but the availability of these tests in health centres were blood group 9 (90 %), RPR 7 (70 %), haemoglobin 9 (90 %), HIV and blood glucose 8 (80 %). Hepatitis B screening test was not available in 2 (66.7) of the hospitals and 4 (40 %) of the health centres (Table 2).

### Functional status of basic FANC laboratory services

In this study the functionality of the available tests as measured by provision of consistence and uninterrupted FANC laboratory in the visited health facilities, the finding showed that 4 (40 %) of health centres reported that haemoglobin and blood glucose tests interpretation for more than a day in the last 1 year. While 1 (33.3 %) of the hospitals reported that haemoglobin and HIV test interpretation for more than a day in the last 1 year.

The most commonly interrupted FANC laboratory tests were RPR and hepatitis B virus screening. RPR test was interrupted in 2 (66.7 %) and 6 (60 %) of the hospitals and health centers respectively. Hepatitis B virus screening test was also interrupted in 2 (66.7 %) of the hospitals and 5 (50 %) of the health centres. Blood group, Rh and urine dipstick (multiple dip stick for Glucose, Albumin and Ketone) tests were not interrupted in all hospitals in the last 1 year, whereas; these tests interrupted in 2 (20 %) of the health centres. Urine microscopy and stool examination not interrupted in all hospitals. While 5 (50 %) of health centres reported that urine microscopy and stool examination interrupted for more than a day in the last 1 year (Table 2).

In general, all health facilities had at least one or more of the basic FANC laboratory tests interruption for more than a day within the last 1 year due to different reasons. Shortage of reagent, equipment failure and electric power disruption were the main causes for the services interruption.

### Laboratory equipment and consumable

Even though basic focused antenatal care laboratory tests do not require high equipment (automated machines) like other laboratory tests; equipment failure and shortage of laboratory supplies were the major problems to deliver FANC laboratory services in most visited health facilities. Among visited laboratories, 5 (38.5 %) had laboratory equipment failure for more than a day in the last 1 year and 7 (53.8 %) had equipment down time for more than a days due reagents stock out, only 9 (69.2 %) of them performed routine preventive maintenance for laboratory equipment and 6 (46.2 %) of the laboratories had equipment maintenances agreement with venders (Table 3). None of the health facilities reported to have laboratory staff trained and designated to maintain laboratory equipment.

**Table 3:** Quality assurances, laboratory equipment and consumable at public health facilities, in Addis Ababa, Ethiopia

Variable	Frequency	Percentage
Laboratories participation on EQA scheme	3	23.1
Having written TAT for all FANC laboratory tests	12	92.3
Having written quality policy manual, safety manual, Sop etc	13	100
Having IQC for all basic FANC laboratory test	4	30.8
Equipment failure more than a day in the last 1 year	5	38.5
Equipment down time more than a day due to stock out	7	53.8
Routine laboratory equipment preventive maintenance performed	9	69.2
Having equipment service agreement with venders	6	46.2

April to May, 2015 (n = 13)

### Quality assurance

From the total 13 visited laboratories only 3 (23.1 %) had participate in External Quality Assurances (EQA) scheme. Except for HIV screening tests, no EQA scheme was established for all FANC laboratory tests in all visited health center laboratories. Among basic FANC laboratory test majority of the tests were performed without internal quality control during study period. The reasons given by providers who working in the visited laboratories for not including internal quality control when performing tests were lack of quality control materials.

One of the providers in the in-depth interview explained that, *“Suppliers (local pharmaceutical agency) not respond timely if regent and other supplies requested from health facilities, because most laboratory commodities come from out*-*country vendor. Therefore most reagents especially quality control materials not available throughout the year for some FANC laboratory tests like blood groups and Rh tests, these affect the quality of services”.* (Male, age 26, laboratory technologies).

All visited laboratories had quality assurance manual (quality laboratory manual), SOPs and other materials that aid laboratory service providers as reference materials in their day to day activities. About 92.3 % of the visited laboratories had established TAT for all FANC laboratory tests (Table 3).

### Human resources, training and motivation

In this study we assessed the availability of the laboratory staffs and their training and motivation status. The standard of laboratories professional expected to be available at each health facility level in Addis Ababa city administration were 8 and 22 laboratory professionals for health centre and hospital, respectively. A total of 123 laboratory personnel were available in all the surveyed health facilities; health centers fulfilled 77.5 % (62/80), whereas hospitals had full filled 92.4 % (61/66) of laboratory staff in respective to their standards. The number of laboratory staffs available in the health centres ranged from 6 to 8 and in hospital from 13 to 21. About 65 % of laboratory services providers had more than 2 years’ experience of work on laboratories services (Table 4). None of the providers had taken training related to FANC laboratory tests.

**Table 4:** Human resources and training status at public health facilities, in Addis Ababa, Ethiopia

Human resources	Health facility type	Numbers of staffs available	Working experiences		Training status on	
			<2 year	>2 year	FANC laboratory	Equipment maintenance
Laboratory technician (Diploma)	Health center	38	12	26	0	0
	Hospital	25	7	18	0	0
Total		63	19	44	0	0
Laboratory technologist (BSC)	Health center	24	9	15	0	0
	Hospital	34	12	22	0	0
Total		58	21	37	0	0
Laboratory technologist (MSC)	Health center	0	0	0	0	0
	Hospital	2	2	0	0	0
Total		2	2	0	0	0

April to May, 2015

Laboratory services providers were claimed to be overloaded by work and poorly motivated, a participant in the in-depth interview said that, “*Motivational aspects and an incentives to the providers were not addressed, even recognition scheme like certificate of appreciation to our work not in place”.* (Male, age 33, laboratory technologist).

## Discussion

This study result shows that only 5 (38.5 %) out of 13 visited health facilities provided all types of basic FANC laboratory investigation. HIV test kits were available in 11 (84.6 %) facilities during the study period. When this finding was compared with the finding of south-western part of Tanzania that had a result of 91–100 % [16], it was lower. Haemoglobin test was available in the 92.3 % of the health facilities. Urine test (multiple dip stick for Glucose, Albumin and ketone) was available in all (100 %) health facilities. This finding was higher than the study which was conducted in public health facilities of Bahir-Dar special zone and south-western part of Tanzania [9, 16].

In this study the availability of RPR test accounts 76.9 %. This is equivalent with the study finding in hospitals and regional laboratories in Ethiopia it accounts 76.5 % [6] and lower than the study which was conducted in public health facilities of Bahir-Dar special zone, which had a result of 25 % [9]. The availability of Hepatitis B screening test was 53.8 %, which is higher when it is compared with the study conduct in hospitals and regional laboratories in Ethiopia that had a result of 20.6 % [6]. The difference might be due to this study was conducted during the time were there is high attention for maternity services due to MDGs.

Regarding to the functionality of FANC laboratory services, all visited health facilities had laboratory services interruption at least one or more of the basic FANC laboratory tests for minimum of a day within the last 1 year. This finding was supported by other studies [8, 9, 16, 17], study conducted in Jimma identified that, urine, blood group and RPR tests were not done to lack of reagents, women who did not have other laboratory tests than the HIV test were more likely to report being not satisfied with services [17]. Similarly Ethiopia Mini Demographic and Health Survey 2014 report showed that among women with antenatal care, nearly 41 % of the ANC clients haven’t their urine tested, 35 % haven’t their blood tested [8].

Regarding laboratory safety issue, 100 % of the visited laboratories had safety manual, personal protective equipment and using incinerators for solid waste disposal. This finding was higher when it is compared with the study conducted to assess the status of HIV screening laboratories in Ethiopia that had a result of 29, 45.2 and 55 % respectively [13]. The difference might be due to trainings on the area of safety precaution. The other possible reason could be this study conducted during the time was Addis Ababa city administration health bureau implement Stepwise laboratory Improvement Process Towards Accreditation (SLIPTA) in most public health facilities. All of visited health facility laboratories liquid waste coming out of the laboratory was released to the sewerage system without any pre-treatment with chemicals or other sterilization techniques. This finding is lower when it compared with the study did to assess the status of HIV screening laboratories in Ethiopia; it accounts 58.5 % [13].

Most of the laboratory services are need electric power supply, unless frequent or prolonged electric power disruption greatly affects health service provision. This study assessed the availability of back-up electric power supply and water in the laboratories, 8 (61.5 %) had generator and 84.6 % had adequate water supply this finding were consistent with the study which conducted to assess the status of HIV screening laboratories in Ethiopia, that had a result of 95.5 % the facilities had back-up electric power and waters supply [13].

Routine preventive maintenance of laboratory equipment were performed in 9 (69.2 %) visited health facilities and 6 (46.2 %) of the laboratories had equipment services agreement. This finding was support by the finding of study done to assess the status of HIV screening laboratories in Ethiopia which identified equipment maintenance as a major problem in supervised laboratories.

## Conclusions

Majority of the health facilities reported incomplete FANC laboratory investigations. Hepatitis B screening test was the lowest available and frequently interrupted test in the study facilities. Furthermore; all visited health facilities had at least one or more basic FANC laboratory tests interruptions more than a day within the last 1 year due to shortage of reagent and electric power disruption. Compared to hospital, in the health centre the availability and functionality of FANC laboratory services were low. None of the health centres had participated in external quality assurance except for HIV test, which might be affect the quality of services.

The findings suggested that providing incomplete FANC laboratory service, supply shortage and electric power disruption in the facilities’ are the major challenge to screening pregnant mothers for pregnancy related health conditions. Since such conditions may affect the outcome of pregnancy, therefore, from the administrators of health facilities and other responsible body extensive efforts should be targeted to avoid services interruption by taking improvement measures including the fulfilment of all FANC laboratory resources.

## Abbreviations

ANC: ante natal care
FNAC: focused ante natal care
EQA: external quality assessment
HGB: haemoglobin
HIV: human immune virus
IQC: internal quality control
RPR: rapid plasma reagin test
SPSS: Statistical Package for Social Sciences
STI: sexually transmitted infections
TB: tuberculosis
TAT: turnaround time
WHO: Word Health Organization

## References

[1] WHO. Maternal mortality fact sheet. Geneva: WHO; 2010.

[2] Jean BN, Francis K, Ali AY, Jason M. Strengthening public health laboratories in the WHO African region: a critical need for disease control. WHO, Regional Office for Africa; 2010.

[3] World Health Organization (2003). Antenatal care in developing countries: promises, achievement and missed opportunities: an analysis of trends, levels and differentials 1999–2001.

[4] WHO. Antenatal care randomized trial: manual for the implementation of the new model. Geneva: WHO; 2002.

[5] Olesegun PA (2012). Improving laboratory service and work force in rural health facilities. J Pak Med Sci.

[6] Belete T, Hailu M, Afework K, Dessalegn T, Negussie G, Wegene T (2004). Laboratory services in hospitals and regional laboratories in Ethiopia. Ethiop J Health Dev.

[7] Assessing progress towards the millennium development goals Ethiopia. MDGs Report; 2012.

[8] Ethiopia Mini Demographic and Health Survey. Addis Ababa; 2014.

[9] Tadesse E, Mirkuzie W, Yibeltal K (2013). Quality of antenatal care services at public health facilities of Bahir-Dar special zone, Northwest, Ethiopia. BMC Health Serv Res.

[10] Federal Ministry Of Health: health and health-related indicators report for EFY 2003. Addis Ababa: Federal Ministry of Health; 2009.

[11] Dereb G, Biadgilegn S, Trivelli M, Hundessa G, Robi ZD, Gerbre-Mariam M, Mekonnen M (2014). Determinant And outcome of early diagnosis of HIV infection among HIV—exposed infants in Southwest Ethiopia. BMC Res Notes.

[12] Abebaw GW, Alemayehu WY, Mesganaw FA (2013). Availability and components of maternity services according to providers and users perspectives in North Gondar, northwest Ethiopia. Reprod Health.

[13] Tegbaru B, Melese H, Tamene W, Gezahegn N, Ahmedin Z, Birhanu H, Tesema D, Messele T (2002). The status of HIV screening laboratories in Ethiopia: achievements, problems encountered and possible solutions. Ethiop J Health Dev.

[14] Central Statistical Agency Of Ethiopia (CSA): summary and statistical report of the 2007 population and housing census. Addis Ababa; 2008.

[15] Population affairs coordination sub process finance and economic development Bureau report, A. A population images; 2009.

[16] As Nyamtema, AB Jong, Urassa DP, Hagen JP, Roosmalen JV (2012). The quality of antenatal care in rural Tanzania. BMC Pregnancy Childbirth.

[17] Villadesen SF, Britt PT, Negussie D, Abebe G, Tilahun A, Friss H, Rasch V. Antenatal care strengthening in Jimma, Ethiopia: a mixed-method needs assessment. J Environ Public Health. 2014. http://dx.doi.org/10.1155/2014/945164. 10.1155/2014/945164 PMC4166433 25258631

